# Baseline Motor Impairment Predicts Transcranial Direct Current Stimulation Combined with Physical Therapy-Induced Improvement in Individuals with Chronic Stroke

**DOI:** 10.1155/2020/8859394

**Published:** 2020-11-25

**Authors:** Adriana Baltar, Daniele Piscitelli, Déborah Marques, Lívia Shirahige, Kátia Monte-Silva

**Affiliations:** ^1^Applied Neuroscience Laboratory, Universidade Federal de Pernambuco, Recife, Pernambuco, Brazil; ^2^School of Medicine and Surgery, University of Milano Bicocca, Milano, Italy; ^3^School of Physical and Occupational Therapy, McGill University, Montreal, Canada

## Abstract

Transcranial direct current stimulation (tDCS) can enhance the effect of conventional therapies in post-stroke neurorehabilitation. The ability to predict an individual's potential for tDCS-induced recovery may permit rehabilitation providers to make rational decisions about who will be a good candidate for tDCS therapy. We investigated the clinical and biological characteristics which might predict tDCS plus physical therapy effects on upper limb motor recovery in chronic stroke patients. A cohort of 80 chronic stroke individuals underwent ten to fifteen sessions of tDCS plus physical therapy. The sensorimotor function of the upper limb was assessed by means of the upper extremity section of the Fugl-Meyer scale (UE-FM), before and after treatment. A backward stepwise regression was used to assess the effect of age, sex, time since stroke, brain lesion side, and basal level of motor function on UE-FM improvement after treatment. Following the intervention, UE-FM significantly improved (*p* < 0.05), and the magnitude of the change was clinically important (mean 6.2 points, 95% CI: 5.2–7.4). The baseline level of UE-FM was the only significant predictor (*R*^2^ = 0.90, *F*_(1, 76)_ = 682.80, *p* < 0.001) of tDCS response. These findings may help to guide clinical decisions according to the profile of each patient. Future studies should investigate whether stroke severity affects the effectiveness of tDCS combined with physical therapy.

## 1. Introduction

Transcranial direct current stimulation (tDCS) is an emerging technique with the potential to enhance the effect of therapeutic approaches in post-stroke rehabilitation [1, 2]. According to the interhemispheric competition model [3, 4], anodal tDCS is applied to increase the excitability of the lesioned hemisphere. In contrast, cathodal tDCS is applied to decrease the excitability of the nonlesioned hemisphere. Lastly, bihemispheric tDCS involves anodal and cathodal tDCS applied simultaneously [5].

Regarding the effects of each tDCS method, it is suggested that bihemispheric tDCS has a more significant effect on chronic stroke [6–8]. Moreover, the positive effect of each tDCS approach on stroke motor recovery has been elucidated by previous studies [9–13]. Notably, recent systematic reviews reported the improvement of upper limb (UL) sensorimotor functions and improvement of activities of daily living following tDCS in post-stroke individuals [8–10, 14].

Despite its great potential, post-stroke subjects show different responses to tDCS. Furthermore, the variability of tDCS effectiveness limits its implementation as standard patient care [15]. A better understating of individual characteristics for predicting motor recovery in responding to treatment should be considered a crucial component for post-stroke rehabilitation.

Following a stroke, neural reorganization, due to spontaneous recovery or induced by therapeutic interventions, is influenced by clinical and biological factors [16–18]. Some of these factors might help to predict therapy-mediated motor recovery [18–21], i.e., stroke chronicity [22, 23], sex [24, 25], age [23, 26], prestroke hemispheric dominance [18], and time since stroke [17].

Initial motor impairment can also predict motor outcomes [27]. Post-stroke motor recovery is highly variable [15], and individuals could present mild to severe motor impairment [28]. Overall, the initial (i.e., baseline) motor impairment is a strong predictor of functional improvement; e.g., moderate motor impairment is associated with better recovery than severe impairment in post-stroke survivors [29].

Notably, previous studies employing tDCS combined with physical therapy included patients with different motor impairment levels and reported heterogeneous results [30–32]. The variability of tDCS response could be related to different aspects related to the technique or the patient's characteristics. Regarding the tDCS, the parameters of the technique, the ideal number of sessions, and the most appropriate stimulation site (lesioned hemisphere, nonlesioned hemisphere, or both hemispheres) should be considered. Concerning the post-stroke individuals, it is important to consider the motor impairment, the location and size of the lesion, and the previous condition of the subject. The most appropriate supporting therapy should also be considered. The heterogeneous results could be related to one or more of these factors (reviewed in Simonetta-Moreau [33]).

Considering predictive factors that might guide stroke recovery, recent studies suggest the development of algorithms or models to determine functional recovery following rehabilitation in either acute or chronic post-stroke individuals [5, 34]. Although there is an increasing number of studies using tDCS in stroke rehabilitation and its relevance for clinical practice, it is unknown whether personal factors, e.g., age and sex, may predict the magnitude of the effect of tDCS on functional recovery [33]. Moreover, UL sensorimotor impairments (e.g., disrupted interjoint coordination, spasticity, and loss of dexterity) are common after stroke and persist in the chronic stage [35, 36]. These deficits may lead to decreased quality of life and social participation. Thus, this study was aimed at investigating if clinical and biological characteristics might predict the tDCS plus physical therapy effects on UL motor recovery in chronic stroke individuals. This knowledge might help to guide clinical decisions according to the clinical profile of each patient as well as to enhance clinical evidence-based practice for neurorehabilitation.

## 2. Methods

### 2.1. Design and Sample

This study is a secondary analysis of data in previously published studies [37, 38] and two ongoing studies (NCT03446378 and NCT02166619) developed at the Applied Neuroscience Laboratory (Universidade Federal de Pernambuco, Brazil).

The local ethics committee approved these studies, and all participants gave written informed consent. Each study was a double-blind (see Intervention), sham-controlled randomized clinical trial. Individuals aged >18 years were included if they presented the following criteria: (i) ischemic or hemorrhagic chronic stroke (≥3 months after onset), (ii) UL sensorimotor impairment due to stroke, and (iii) no cognitive impairment according to the Mini-Mental State Examination [39] and being able to perform some movement with the wrist and/or thumb. Exclusion criteria were as follows: spasticity at the wrist > 3 according to the Modified Ashworth Scale [40], aphasia, or any contraindications for tDCS, according to safety guidelines [41, 42]. Eighty chronic post-stroke subjects who received active tDCS treatment were analyzed.

### 2.2. Intervention

Participants were randomly assigned to the tDCS protocol group: anodal on the lesioned motor cortex (1 mA/13 min or 2 mA/20 min), cathodal on the nonlesioned motor cortex (1 mA/9 min or 2 mA/20 min), or bihemispheric tDCS (2 mA/20 min). The lesioned and nonlesioned motor cortex (C3/C4) was determined according to the 10/20 reference system [43]. For anodal and cathodal tDCS, the reference electrode was placed over the contralateral supraorbital area. In all trials, randomization was performed by an independent investigator not involved in any of the research phases through the website http://www.randomization.com.

All participants received ten to fifteen sessions of tDCS (3 to 5 times/week) plus usual-care physiotherapy (45 minutes to 1 hour). Physiotherapy consisted of constraint-induced movement therapy, virtual reality, or task-oriented exercises. All participants attended physical therapy sessions after tDCS. All subjects were evaluated at the baseline and after the completion of all tDCS sessions plus physical therapy (see Outcome Measurement).

Assessors (pre and post) and participants were blind to the tDCS protocol. A not-involved researcher was responsible for the application of tDCS. The allocation concealment was met using opaque sealed envelopes, which were stored in a locked room.

### 2.3. Outcome Measurement

The upper extremity section of the Fugl-Meyer scale (UE-FM) was used to measure sensorimotor impairment in post-stroke survivors [44, 45]. The total score ranges from 0 to 66; higher scores indicate better motor function [44]. In chronic stroke individuals, the minimal clinical important difference (mCID) ranges from 4.25 to 7.25 [46].

### 2.4. Data Collection

Biological (age, sex) and clinical (time since stroke, brain lesion side: dominant or nondominant according to brain dominance, determined by self-reported handedness) characteristics were collected for each participant. UE-FM scores at the baseline and after all the tDCS sessions plus physical therapy were also collected.

### 2.5. Statistical Analysis

Descriptive statistic was used to present clinical and biological characteristics. Data were checked for normal distribution (i.e., Shapiro-Wilk test *p* value > 0.05 and by visual inspection of a quantile-quantile plot).

#### 2.5.1. Preliminary Data Analysis

Before subjecting the data to regression models, several analyses were run to control for potentially confounding baseline factors. In particular, in order to identify baseline differences between the three tDCS protocols, age, time since stroke, and UE-FM scores were submitted to one-way ANOVAs. Chi-square (*χ*^2^) tests were used to assess the difference between tDCS protocols for sex, handedness, and brain lesion side. To investigate the difference in the UE-FM scores at baseline and post-treatment within the entire cohort, paired Student's *t*-test was used, and 95% confidence intervals (CI) of mean change were reported. Finally, one-way ANOVAs were used to investigate between-group differences in UE-FM scores at post-treatment and in UE-FM changes across the three tDCS protocols. In case of significant effects, pairwise contrasts with Bonferroni corrections were used.

#### 2.5.2. Regression Models

In order to analyze the influence of clinical and biological variables on post-stroke motor recovery, a multiple linear regression was performed. Post-treatment UE-FM was considered a dependent variable. Independent factors included in the model were variables that had previously been identified as associated with tDCS response: age and sex, time since stroke, brain lesion side, and baseline motor impairment [47, 48]. A backward stepwise regression (entry criteria: *p* ≤ 0.05; removal criteria: *p* ≥ 0.10) was used to find the best fit. Before performing multiple regression, independent variables were tested for multicollinearity (i.e., strong correlations among predictor variables, Pearson correlation coefficient (*r*) greater than 0.7), homoscedasticity, and outliers. Eighty subjects were considered an adequate sample size for regression analyses [49, 50].

SPSS version 21 (IBM, Armonk, NY, USA) was used for the statistical analysis, and the level of significance was set at *p* < 0.05.

## 3. Results

tDCS plus physical therapy was administrated to all participants (*n* = 80). Individuals submitted to cathodal, anodal, and bihemispheric tDCS were 34% (*n* = 27), 47% (*n* = 38), and 19% (*n* = 15), respectively. The biological and clinical characteristics of participants are presented in Table 1 (see baseline variables).

At baseline, one-way ANOVAs and chi-square (*χ*^2^) tests showed no differences (*p* > 0.05) between the three groups for age, time since stroke, UE-FM scores, sex, handedness, and brain lesion side, respectively. Tests are presented in Table 1.

All participants showed a significant improvement in UE-FM scores after treatment (*t*‐test_(79)_ = 11.57, *p* < 0.001). Moreover, the UE-FM mean change was clinically important (6.2 points, 95% CI: 5.2–7.4). Post-treatment UE-FM scores are shown in Table 1. No differences were found on the UE-FM score at post-treatment (*F*_(2, 77)_ = 2.732, *p* = 0.071) and on UE-FM score changes (*F*_(2, 77)_ = 1.171, *p* = 0.315), between the three tDCS protocols.

All assumptions for multiple regression were met. Stepwise regression showed that only baseline UL impairment was a significant predictor of changes in UE-FM scores after tDCS plus physical therapy (*R*^2^ = 0.90, *F*_(1, 76)_ = 682.80, *p* < 0.001). The results of the stepwise regression are shown in Table 2.

## 4. Discussion

The ability to assign the right patient to tDCS therapy would permit one to make a rational decision to add it to rehabilitation programs. Our findings showed that the baseline UL impairment might predict tDCS-induced recovery. We found significant *R*^2^ = 0.90; i.e., 90% of the variance in post-treatment UE-FM scores can be predicted from the baseline UE-FM score. In particular, we found a positive regression coefficient (*β* = 0.95) indicating that as the value of the independent variable increases (i.e., baseline UE-FM score), the mean of the dependent variable also tends to increase (i.e., UE-FM score after treatment).

Although limited for the control group's absence, our results are in line with previous studies [10, 14, 51, 52]; i.e., tDCS plus physical therapy shows a positive effect on UL motor recovery. Moreover, we demonstrated that chronic patients reached a clinically relevant improvement after tDCS plus physical therapy regardless of tDCS protocols, age, sex, times since stroke, and brain lesion side.

This result confirms previous studies by showing that tDCS combined with other therapies induces UL recovery in patients with stroke [7, 37, 38, 53].

### 4.1. Predictive Factor of Recovery following tDCS

In agreement with our findings, studies [19, 26] provided evidence that initial motor impairment, commonly measured with the UE-FM, predicts functional outcomes in patients with stroke. In general, greater baseline impairment is associated with worse motor outcomes [54, 55]. However, to our knowledge, no previous study has investigated factors influencing functional UL recovery following tDCS.

One of the most common measures studied to predict UL stroke recovery is motor evoked potential (MEP) elicited with transcranial magnetic stimulation (TMS). To date, there is increasing evidence about the usefulness of TMS to study the activation and structural integrity of ipsilesional motor networks for predicting and improving motor recovery [56–59]. Indeed, studies have reported that MEP measurement had higher predictive power than clinical outcome assessment [60, 61]. However, TMS is not always available in clinical environment TMS is generally few accessible and may be influenced by several factors [62], limiting its implementation in clinical practice. Therefore, the use of clinical makers such as the Fugl-Meyer scale to predict tDCS response at the individual level might be more feasible for routine clinical use.

Future studies are needed to address the predictive power and reliability of the Fugl-Meyer scale compared with MEPs as a marker to predict motor recovery in chronic stroke following tDCS treatment. However, the prediction of tDCS responders from non-responders in chronic post-stroke individuals might be more challenging than that in the acute/subacute phase since other factors are involved, e.g., biomechanical factors [63], psychological factors, and changes in brain structural and/or functional connectivity [64]. Thus, to take into account the complexity of motor recovery in the chronic phase, predictive models should include both clinical and neurophysiological biomarkers [21]. Indeed, a recent guideline and systematic review suggest that for a proper selection of post-stroke subjects for tDCS, assessment of anatomo-functional parameters and initial motor impairment should be considered [2, 65].

### 4.2. Nonpredictive Factors of Recovery following tDCS

Age and sex were not significant factors limiting tDCS-induced motor UL recovery. Also, previous studies have demonstrated motor recovery induced by various therapies regardless of age and sex [20, 66]. Besides, some evidence [67, 68] suggested that noninvasive brain stimulation- (NIBS-) induced plasticity is decreased with age, although some other studies are in line with our findings reporting no age-related effects [69, 70]. The tendency of elderly patients to experience more severe strokes with greater motor impairment [71] should be considered to avoid misinterpretation of aging as a predictive factor in stroke recovery. Following the same reasoning, higher frequency of severe strokes in women [24] reflecting worse motor impairment could contribute to sex-related differences in the motor outcome following NIBS. Indeed, sex differences on functional outcomes after stroke disappear after adjustment for confounding factors such as stroke severity [72].

Although our regression did not find that the brain lesion side was a significant predictor for motor recovery, a previous study found it [73]. These authors suggested that the affected UL motor recovery is dependent on brain dominance of the impaired hemisphere. Increasing evidence suggests that interhemispheric inhibition is influenced by brain dominance and in individuals with stroke is greater when the non-dominant hemisphere is affected [74]. Along with the lesion side, other factors also influence motor recovery, such as type of stroke, lesion location (i.e., cortical or subcortical), and size [33].

Even though our first aim was to investigate predictive factors of tDCS effects on UL motor recovery in chronic stroke patients, we also reported novel findings regarding tDCS protocol comparison. Few studies have routinely investigated the bilateral (i.e., bihemispheric tDCS) versus unilateral (i.e., anodal or cathodal tDCS) similarity efficacy in changing paretic UL performance. We found no significant difference among the three tDCS protocols on UE-FM score improvement, suggesting a nondependent effect of tDCS protocol stimulation on UL recovery. In contrast, O'Shea et al. [75] have reported the superiority of anodal and cathodal over bihemispheric tDCS in speeding reaction time in chronic stroke patients. The current intensity used in our bihemispheric tDCS protocol (2 mA), or multiple sessions versus one of O'Shea et al.'s study, could explain the different findings.

Apart from the heterogeneity of tDCS parameters, the similarities seen between the tDCS groups could be related to motor impairment levels. Previous studies have suggested that individuals with mild or moderate impairment showed considerable activity in the lesioned hemisphere and/or partial integrity of the corticospinal tract [76, 77]. In light of this physiological finding, we can hypothesize that for a mild to moderate severity population, it is favorable to increase the activity present in the lesioned hemisphere, rather than inhibit the nonlesioned one. On the other hand, it is also known that patients with severe motor impairment present greater activity in the non-lesioned hemisphere [27], which could also promote negative motor-related consequences [78, 79]. Accordingly, using the cathodal tDCS to reduce the activity in the nonlesioned hemisphere could promote sensorimotor gains. Thus, the lack of difference between the three groups of tDCS might be due to different motor impairment levels across participants.

In line with our results, by comparing the effectiveness of repetitive TMS on motor recovery in relation to the time from stroke, the review of Dionísio et al. [80] also did not detect that repetitive TMS effectiveness differs among acute, subacute, or chronic phase, suggesting that time since stroke does not affect NIBS-induced effect on motor recovery. However, it is important to highlight that the time of tDCS therapy after the stroke onset could significantly influence the efficacy of a given tDCS protocol [81]. For example, based on the classical concept of interhemispheric competitive interaction (reviewed in Nowak et al. [3]), it is expected that cathodal tDCS may provide beneficial effects for some patients by reducing contralesional hemisphere activity. On the other hand, the effects may be detrimental for other subjects, depending on the individual's significance of the contralesional activity in controlling the paretic movement. This issue is still unclear and needs to be addressed in further studies.

Some limitations should be considered in this study. First, our sample size is reduced, and the results should be interpreted with caution since there is no equal distribution, considering the sex and age group. Second, our data did not include the lesion volume/site, and this could limit the interpretation of our findings since individuals with cortical or subcortical lesions could respond differently [33]. Finally, it is important to highlight that all patients underwent physical therapy, and this could influence the results since physical therapy is well established to promote motor recovery [82]. Besides, the changes in motor function may spontaneously occur after stroke. However, it is suggested that for better recovery, larger doses of physical therapy may be required to promote improvements [83]. tDCS could act as priming to enhance the effects of physical therapy [84]. Moreover, this study is a secondary analysis of previous works that showed how tDCS increased the therapy effect.

Despite the positive effects of tDCS on motor recovery [9, 10, 51], several scientific issues remain unresolved. Studies are warranted to investigate the dose-response relationship and to profile patients who might potentially benefit from tDCS.

## 5. Conclusion

To date, no precise indicators are available to predict positive effects following tDCS plus physical therapy on UL recovery. Our results suggest that a simple metric of baseline motor impairment by means of UE-FM may be predictive for clinical motor improvement induced by tDCS. Overall, this knowledge may help to guide clinical decisions according to the profile of each patient, reducing tDCS therapy failure and making it practically useful in clinical settings. Future studies should consider the motor impairment of post-stroke individuals to investigate personalized protocols of tDCS.

## Figures and Tables

**Table 1:** Clinical and demographic characteristics.

Participant characteristics	Cathodal tDCS (9-20 min; 1-2 mA, *n* = 27)	Anodal tDCS (13-20 min; 1-2 mA, *n* = 38)	Bihemispheric tDCS (20 min; 2 mA, *n* = 15)	Between-group differences
Baseline				
Age (in years)	60.5 (±9.9)	56.6 (±.9.2)	59 (±7.8)	*F* = 1.40, *p* = 0.253^∗^
Sex, *n* (female/male)	27 (11/16)	38 (13/25)	15 (6/9)	*χ* ^2^ = 0.336, *p* = 0.845^#^
Handedness, *n* (right/left)	27 (24/3)	38 (38/0)	15 (14/1)	*χ* ^2^ = 4.211, *p* = 0.122^#^
Time since stroke (in months)	31.1 (±26.8)	36.7 (±28.9)	41.2 (±27.9)	*F* = 0.659, *p* = 0.520^∗^
Brain lesion side, *n* (dom/non-dom)	27 (16/11)	38 (20/18)	15 (7/8)	*χ* ^2^ = 0.652, *p* = 0.722^#^
UE-FM score	27.7 (±15.7)	30.6 (±15.5)	37.9 (±11.3)	*F* = 2.262, *p* = 0.111^∗^
Post-treatment				
UE-FM score	32.9 (±15.2)	37.7 (±14.6)	43.9 (±14.2)	*F* = 2.732, *p* = 0.071^∗^

Values are mean and standard deviation, except for sex, time since stroke, and lesion side (count). tDCS: transcranial direct current stimulation; UE-FM: upper extremity Fugl-Meyer scale; dom = dominant; non-dom = nondominant. ^∗^One-way ANOVA; ^#^Chi-square test.

**Table 2:** Results of the regression analyses.

Model	Variables	*β*	(SE)	*β* stand	*t*	*p*	*R* ^2^	*R* ^2^ change
1	Age	0.04	0.06	0.02	0.66	0.51		
	Sex	-0.24	1.18	-0.01	-0.21	0.84		
	Time since stroke	0.01	0.02	0.01	0.28	0.78		
	Brain lesion side	0.91	1.16	0.03	0.79	0.43		
	Baseline UE-FM	0.96	0.04	0.96	24.94	<0.001	0.902	0.902

2	Age	0.04	0.06	0.03	0.69	0.49		
	Time since stroke	0.01	0.02	0.01	0.31	0.76		
	Brain lesion side	0.89	1.15	0.03	0.77	0.44		
	Baseline UE-FM	0.96	0.04	0.96	25.12	<0.001	0.902	<0.001

3	Age	0.04	0.06	0.03	0.69	0.49		
	Brain lesion side	0.85	1.14	0.03	0.75	0.46		
	Baseline UE-FM	0.96	0.04	0.96	25.33	<0.001	0.901	<0.001

4	Brain lesion side	0.93	1.13	0.03	0.82	0.41		
	Baseline UE-FM	0.96	0.04	0.96	25.54	<0.001	0.901	-0.001

5	Baseline UE-FM	0.95	0.04	0.95	26.13	<0.001	0.900	-0.001

UE-FM = upper extremity Fugl-Meyer scale; SE = standard error. Note that only baseline UE-FM is a significant predictor in the regression models.

## Data Availability

The data that support the findings of this study are available from the corresponding author (DP) upon reasonable request.
